# The Design and Relevance of a Computerized Gamified Depression Therapy Program for Indigenous Māori Adolescents

**DOI:** 10.2196/games.3804

**Published:** 2015-03-03

**Authors:** Matthew Shepherd, Theresa Fleming, Mathijs Lucassen, Karolina Stasiak, Ian Lambie, Sally N Merry

**Affiliations:** ^1^School of Counselling, Human Services and Social WorkDepartment of EducationUniversity of AucklandAucklandNew Zealand; ^2^Werry Centre for Child and Adolescent Mental HealthDepartment of Psychological MedicineUniversity of AucklandAucklandNew Zealand; ^3^Werry Centre for Child and Adloescent Mental HealthDepartment of Psychological MedicineUniversity of AucklandAucklandNew Zealand; ^4^School of PsychologyFaculty of ScienceUniversity of AucklandAucklandNew Zealand

**Keywords:** computerized cognitive behavioral therapy, Māori, indigenous populations, depression, consumer opinions, participatory design

## Abstract

**Background:**

Depression is a major health issue among Māori indigenous adolescents, yet there has been little investigation into the relevance or effectiveness of psychological treatments for them. Further, consumer views are critical for engagement and adherence to therapy. However, there is little research regarding indigenous communities’ opinions about psychological interventions for depression.

**Objective:**

The objective of this study was to conduct semistructured interviews with Māori (indigenous New Zealand) young people (taitamariki) and their families to find out their opinions of a prototype computerized cognitive behavioral therapy (cCBT) program called Smart, Positive, Active, Realistic, X-factor thoughts (SPARX), a free online computer game intended to help young persons with mild to moderate depression, feeling down, stress or anxiety. The program will teach them how to resolve their issues on their own using Cognitive Behavioural Therapy as psychotherapeutic approach.

**Methods:**

There were seven focus groups on the subject of the design and cultural relevance of SPARX that were held, with a total of 26 participants (19 taitamarki, 7 parents/caregivers, all Māori). There were five of the groups that were with whānau (family groups) (n=14), one group was with Māori teenage mothers (n=4), and one group was with taitamariki (n=8). The general inductive approach was used to analyze focus group data.

**Results:**

SPARX computerized therapy has good face validity and is seen as potentially effective and appealing for Māori people. Cultural relevance was viewed as being important for the engagement of Māori young people with SPARX. Whānau are important for young peoples’ well-being. Participants generated ideas for improving SPARX for Māori and for the inclusion of whānau in its delivery.

**Conclusions:**

SPARX computerized therapy had good face validity for indigenous young people and families. In general, Māori participants were positive about the SPARX prototype and considered it both appealing and applicable to them. The results of this study were used to refine SPARX prior to it being delivered to taitamariki and non-Māori young people.

**Trial Registration:**

The New Zealand Northern Y Regional Ethics Committee; http://ethics.health.govt.nz/home; NTY/09/003; (Archived by WebCite at http://www.webcitation/6VYgHXKaR).

## Introduction

### Adolescent Depression in New Zealand

Depression is a major concern globally and population-based studies internationally have indicated that 12-month prevalence rates are as high as 6.7% in adolescents [1]. There appear to be conflicting rates of depression reported for indigenous young people (Māori, the indigenous Polynesian people of New Zealand), as these vary from being comparable to nonindigenous young people [2] through to being approximately two times higher than in nonindigenous populations [3]. In New Zealand, a recent nationally representative study showed that rates of depression for Māori high school students (13.9%) were comparable to New Zealand European young people (12.1%), with 18.3% of Māori girls and 8.7% of Māori boys reporting depressive symptoms in the clinical range on the Reynolds Adolescent Depression Scale-Short Form (RADS-SF) [4]. However, other studies of depression in New Zealand have shown significantly higher rates for Māori, with 16.4% of Māori girls reporting depressive symptoms in the clinical range on the RADS-SF compared with 12.7% of New Zealand European girls [5].

### Computerized Cognitive Behavioral Therapy

The National Institute for Clinical Excellence in the United Kingdom recommends cognitive behavioral therapy (CBT) as a first line treatment for adolescents with mild to moderate depression [6]. However, access to therapists is often problematic and interest in computerized CBT (cCBT) has increased. Although cCBT has been found to be effective for adults with depression [7-9], it is still considered an emerging therapy for the treatment of depression in children and adolescents [10], with initial results for cCBT among adolescents appearing promising [11,12]. Research has demonstrated that cCBT has the potential to be a low-cost and easily accessible option for those in need of therapy [12].

However, indigenous minority adolescents rarely seek professional face-to-face help as a first treatment option for depression [13]. cCBT has the potential to increase access to therapy for these indigenous youth, if it can be delivered in a way that is acceptable and appealing to these young people.

Ethnic minority populations are largely missing from the efficacy studies that make up the evidence base for psychological treatments. This is often due to the inclusion of small samples, which limit the accuracy of statistical inferences [14-17]. Similarly, few studies capture qualitative information because of the small number of ethnic minority participants in studies to date. Ethnic minority opinions (including the perspectives of indigenous people in colonized countries who are often in a minority) are therefore missing in relation to the development of interventions generally, and cCBT programs in particular.

There are implications for those that design cCBT programs, in that the evidence base that they draw from to inform these therapies is fairly limited for ethnic minority groups, with a review of cCBT for adults highlighting that future research in the field needs to specifically assess the acceptability of cCBT among indigenous minority groups [18]. Thus, one cannot assume that CBT theories will be applicable in the same way that they are for nonindigenous people as they are for indigenous minority groups. There is evidence that CBT can be applicable for ethnic minority groups [15,17], but computerization brings an extra set of challenges. For Māori, for example, there is a strong focus on the value of family, so that a therapy delivered by computer may be less acceptable to the Māori community than in cultures with more of a focus on individuals.

This study provides an opportunity to begin to understand some of these much needed “consumer opinions”. Not surprisingly, there has been little research published on psychological interventions for Māori [19-21]. Hence, there is a dearth of information that has recorded the attitudes and opinions of Māori toward the development of CBT programs [19] and attitudes of tangata whenua (indigenous people of New Zealand) to the development of a cCBT resource have never been captured before. Since the rates for depression are especially high for Māori girls (when compared to international rates) [1], it is imperative that Māori have input into interventions that will be developed and trialled, for their benefit. An approach to research, which actively involves Māori, can also serve as a potential model for research with other indigenous minority groups.

In New Zealand, Smart, Positive, Active, Realistic, X-factor thoughts (SPARX), which is a form of cCBT, has been funded by the Prime Minister’s Youth Mental Health Project, and this form of cCBT has been made available free of charge to anyone wanting to access the program on a national level since April 2014.

### Smart, Positive, Active, Realistic, X-Factor Thoughts Computerized Cognitive Behavioral Therapy Program

SPARX (Smart, Positive, Active, Realistic, X-factor thoughts) is a free online computer program intended to help young persons with mild to moderate depression, feeling down, stress or anxiety. Through the game, the program teaches them how to resolve their issues on their own, using Cognitive Behavioural Therapy. Based in a 3D fantasy world, the game leads players through seven realms (each lasting between 30 and 40 minutes). In the beginning of SPARX, the user meets the Guide who explains what SPARX is and how it could help. Then the user customizes an avatar and starts to journey within the seven provinces in order to complete different quests. In the first level, gamers challenge GNATS (Gloomy Negative Automatic Thoughts). These GNATS fly towards the avatar and say negative things like, for example: “you're a loser”. Further in the game, the user meets different characters, solves puzzles and completes mini games. As soon as a quest is completed, the Guide explains how to use new skills in order to feel better, solve problems and enjoy real life. Players complete one or two levels in the game each week, during three to seven weeks.

When we developed SPARX, our aim was to design it to appeal to all young people in New Zealand, regardless of ethnicity [11]. We utilized a participatory design approach, with input throughout from young people of all ethnicities, from CBT clinicians and from advisors from the major ethnic groups in New Zealand. This provides a potential model for the collaborative development of cCBT programs. The program’s development included: a Māori cocreator (MS); input from Māori CBT experts; cultural guidance from a kaumātua (respected elder); and the computer game development company was led by a Māori woman. In the design of SPARX, we attempted to incorporate Māori symbolism within the intervention, albeit within a more general fantasyland format, and to deliver the intervention in a way we believed would resonate with Māori. Having incorporated these elements, we carried out this study to investigate taitamariki (Māori adolescents) and whānau/family opinions regarding the relevance of SPARX to Māori, including the cultural acceptability of designs and content, and of the perceived relevance of SPARX to Māori. The study took place during the beta testing stage of SPARX’s development; the aim of the study was to obtain user feedback so that the findings could be used to inform the finalized version of the program. This is of international interest because of the lack of information about the relevance of cCBT programs cross-culturally, and to indigenous communities in particular.

## Methods

### Kaupapa Māori Methodology

Western research traditionally holds an individualistic approach to epistemology. However, the traditional Māori perspective has been to view the world in a collectivist way. Māori culture places an emphasis on the individual acting in a way that would seek to put the whānau (extended family) and iwi (tribe) needs before their own needs. This has implications for research from a Māori worldview, and this way of carrying out studies has come to be known as Kaupapa Māori research [22,23].

Kaupapa Māori research has emerged over the past two decades, alongside an increasing awareness and acknowledgement from academia of Māori epistemology, coupled with Māori ways (tikanga customs and protocols) of conducting research. Kaupapa Māori research encompasses an analytical approach that is about thinking critically, which includes critiquing Western definitions and constructions of Māori people and their worldview. It is also about valuing Māori self-determination and encouraging Māori participation in the research process [22]. Kaupapa Māori research does not exclude the use of other research methods, but seeks to integrate these in a culturally sensitive way that is beneficial for Māori [21].

Western psychological models such as CBT have tended to focus on an individual’s internal psychological state, for example, a change in one’s thoughts and feelings leads to improved mental health. In contrast, Māori culture emphases the importance of being connected to extended family (whānau), genealogy going back many generations (whakapapa), tribe (hapu and iwi), environment (land, rivers, seas, and mountains), and spiritual (wairua) and physical health [24-28]. Each of these dynamics means that mainstream Western therapeutic approaches might have limited appeal or limited therapeutic power with Māori young people.  cCBT, with its analytic focus on individual thoughts, behaviors, and feelings, without processes of cultural connection, might be considered antithetical to Māori world views. Alternatively, the use of Māori images, the use of the story telling, and opportunities for holistic learning processes utilized in SPARX might have some appeal.

It is important to also note that research that has been conducted in the past has often been detrimental to Māori communities [22]. The history of research within New Zealand has predominantly reflected a distinct patriarchal process in which the Māori worldview has often been marginalized [23]. In this research, we used a Kaupapa Māori approach to ensure that the method of engagement was inclusive of Māori input and respected Māori protocols.

### Epistemological Orientation

The study was led by the first author, MS, who took a critical realist position, which posits that in order to understand the meaning of the data, it is essential to understand the context in which the phenomena takes place and the method by which the data are collected [24]. The first author, MS, is Māori and ensured that all study processes took into account Māori processes and protocols. For example, as suggested by other Kaupapa Māori researchers [26,29], MS was welcomed with a powhiri (formal welcome ceremony) when conducting the focus groups. He responded with a mihi (formal speech), after which waiata (traditional Māori song/s, which had been specially chosen to fit the context) were performed and kai (food) was then shared.

### Participants

In total, seven focus groups were conducted, with a total of 26 Māori participants who were recruited through word of mouth (Table 1). There was no screening of participants, and they did not have to have depressive symptoms to participate. The three types of groups that were conducted were: (1) adolescents; “taitamariki”, (2) “taitamariki mothers”, and (3) family; “whānau”. The taitamariki group was conducted with young people from a kapa haka group (Māori youth traditional performing arts group) and strongly identified as being Māori. This focus group was held at the kapa haka group’s school marae (sacred meeting place). The second type of focus group included young mothers (age 16 to 18 years old). This group was invited to participate because of the high rates of depression among Māori females. The third type of group comprized interviews with whānau (families) that were held in whānau homes. Whānau involvement in all aspects of life, including mental well-being, is important for Māori, and is considered one of the four cornerstones of Māori holistic well-being [30]. The inclusion of feedback from whānau was thus seen as particularly important. Māori protocols (tikanga) were followed when conducting these interviews [29].

**Table 1:** Number and type of focus groups and participants.

Group	Group type	N	n (participants16-18 years)	n (participants parent/caregiver of adolescent)
1	Taitamariki (adolescents)	8	8	-
2	Taitamariki mothers	4	4	-
3	Whānau (family)	4	2	2
4	Whānau	1	-	1
5	Whānau	3	2	1
6	Whānau	3	1	2
7	Whānau	3	2	1

### Procedure

All participants were shown the prototype of SPARX on a computer and asked for feedback during focus groups. A semistructured format was used. The sessions lasted between 30 and 60 minutes. Feedback was sought about the design and applicability of the content of the SPARX program. At the conclusion of the focus group, participants were invited to complete a questionnaire about their views of the session and the prototype SPARX program. The purpose of the questionnaire was to include rating scales so participants had the opportunity to quantify their views.

### Focus Group Questionnaire

The questionnaire contained five questions, which consisted of four Likert scales (on a five point scale), four free-text spaces, and one closed question.

**Table 2:** Focus group questionnaire.

Questions	Rating scale
Q 1. How much were you able to express your opinions in the focus group?	1= “Not at all” 5= “Totally”
Q 2. Overall what did you think about the look and style of the game?	1= “Didn’t like it at all” 5= “Liked it a lot”
Q 3. Overall what did you think about the content (messages and information to help people) in the game?	1= “Didn’t like it at all” 5= “Liked it a lot”
Q 4. Overall what did you think about the cultural content in the game (Māori costume design and building/environment) designs?	1= “Didn’t like it at all” 5= “Liked it a lot”
Q 5. Would you like to make any other comments?	No rating needed

### Numerical Data Analysis

Numerical data were analyzed using descriptive functions of the Statistical Package for the Social Sciences 15.0. The free-text comments were analyzed using a general inductive approach [31].

### Data Analysis

This qualitative research was conducted using a General Inductive Analysis (GIA) [32]. GIA involves collecting the raw data, processing it, and finally interpreting the data to derive concepts and themes from it [33]. GIA fits with the exploratory nature of this research, in that we were seeking to understand participants’ experiences that are not fully understood yet. Inductive methods are also able to put into language peoples’ experiences about the research question that is being put before them [34]. This fits with the aim, which was to gather important data about the design of the cCBT program and then to identify the many contextual variables that may be relevant from the data [35].

The qualitative method utilized for this research was thematic analysis [36]. Thematic analysis was favored over methods such as discourse analysis, as the study aimed to organize and summarize the content of the interviews rather than analyze the way in which participants constructed their own experiences [35].

Braun and Clarke’s [36] six-step process of thematic analysis was used to identify, analyze, and present the main themes from both the focus group and the free-text data using NVivo8 (computer software to conduct qualitative analysis). Initial codes were generated during the first reading of the data, and codes that were similar, but distinct, were kept separate. A second reading of the data confirmed the coding of the themes. An independent researcher read one third of the transcripts and their themes were compared to the themes that MS identified. Any differing opinions about themes were discussed until agreement was reached about which themes to include. By carrying out a qualitative study, we have endeavoured to ensure that the data collection has been exploratory and that preconceptions have been kept to a minimum.

### Ethics

The New Zealand Northern Y Regional Ethics Committee granted approval for this study (NTY/09/003). All participants provided their own written consent. No inducements were offered.

## Results

### Identified Themes

A number of themes were identified from the data, and these have been organized into four categories: (1) computerized therapy has good face validity and is seen as potentially effective and appealing, (2) cultural relevance was viewed as being important for the engagement of Māori young people with SPARX, (3) whānau are important for young peoples’ well-being, and (4) ideas for improvement of SPARX for Māori.

### Computerized Therapy has Good Face Validity and Is Seen as Potentially Effective and Appealing

Taitamariki and whānau generally considered that SPARX could help young people with depression and considered that SPARX would be able to teach skills to help deal with depression.

SPARX is like a computer game that can help with depression*.* Participants acknowledged this assisted with engagement. Participants described SPARX as being a resource that could help adolescents who are experiencing depression.

This game is more about helping you through it (depression) though I just realized it now (in response to MS asking what are your initial thoughts).

Group 6 Female Participant 1

SPARX was able to teach skills. Most participants connected with the characters in SPARX and thought they could teach them skills. A skill that most participants understood and felt motivated to complete was the breathing/relaxation skill.

How everything in there we knew and everything that gets you thinking, especially when they said to breathe. It makes you want to breathe with it and it is only a video game (comment during spontaneous discussion).

Group 3 Male Participant 1

The main thing was probably breathe in, breathe out. It is a good exercise.

Group 2 Female Participant 2

It is good to breathe when you get angry.

Group 2 Female Participant 3

It calms you down (in response to MS asking about what messages did you take away).

Group 2 Female Participant 4

### Cultural Relevance Was Viewed as Being Important for the Engagement of Māori Young People With Smart, Positive, Active, Realistic, X-Factor Thoughts

Most of the participants valued the Māori designs on the characters and there was a range of opinions about whether participants found them relevant. Participants were also interested in the whakapapa (genealogy) of the characters in SPARX. Participants acknowledged that the characters were able to teach skills and wondered whether the Guide character could be a role model for taitamariki.

Māori designs within the SPARX environment were relevant for Māori. Most participants noticed the Māori designs and thought that the inclusion of the designs helped to enhance the engagement and gaming experience of SPARX.

Hey, that is cool...Yes, it snuck up to me. And poutama’s (Māori art design, symbolizing a climb up toward a goal) got a good meaning too. That is really cool. That is like telling them to get happier. So it makes you feel like you have got to try.

Group 5 Female Participant 1

Yes, I was feeling that too (in response to discussion led by MS asking for feedback about the graphic designs in SPARX).

Group 5 Female Participant 2

**Figure 1:**
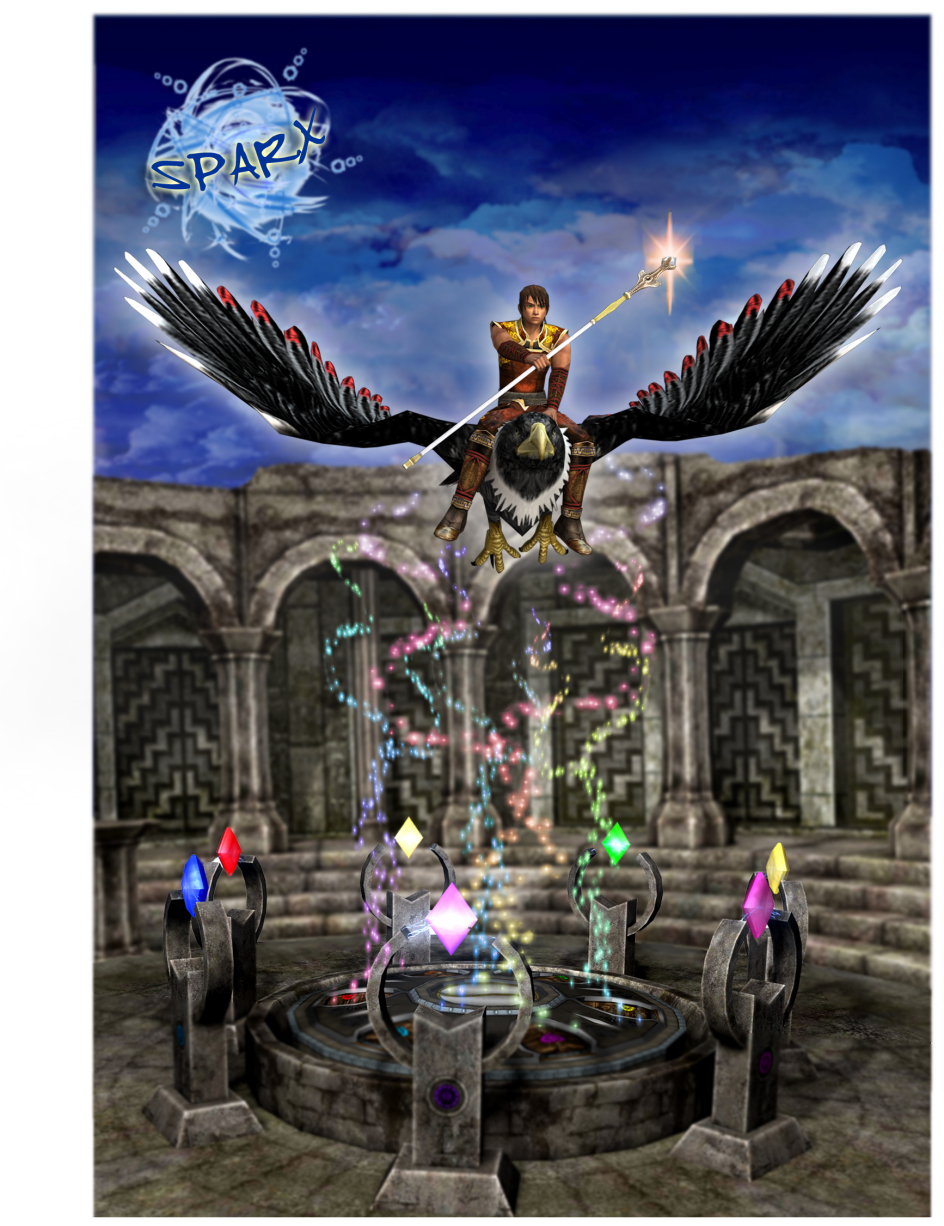
Picture of the poutama (staircase) design.

The participants spoke of the value of Māori designs in SPARX. Most participants in the focus groups thought that the Māori designs on the outfits of the main SPARX characters were a good idea. Most recognized the hybrid nature of the design of the characters (see Figure 1 for an image of a SPARX character), whereby they were seen as being Māori, but existed in a different context, such as a medieval fantasy environment. No participants reported that the Māori designs limited the appeal of the program.

I reckon he looks mean as. He looks like a medieval Māori (in response to MS asking for opinions about the Guide).

Group 5 Female Participant 1

**Figure 2:**
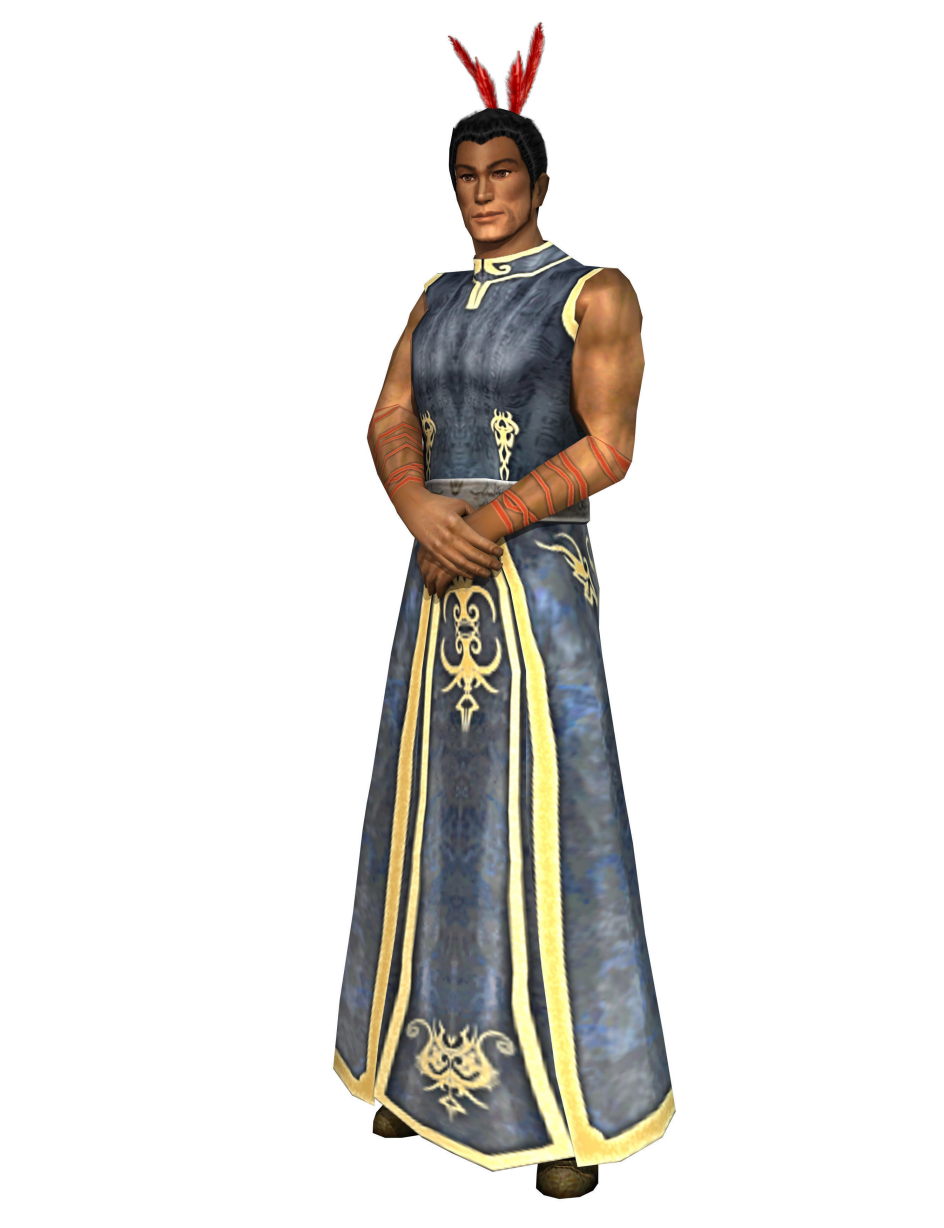
Picture of character from SPARX.

There were some differing views. A small number of participants, both taitamariki and parents/caregivers, did not understand the hybrid concept of the designs (see Figure 2).

It looks like a couple of people from the medieval time that have nothing to do with anything Māori, they don’t really look Māori (spontaneous discussion about what is seen as applicable for Māori).

Group 3 Female Participant 3

It is important that SPARX characters include their whakapapa (genealogy)*.* Participants thought it was important that the characters include their whakapapa in the SPARX program. For example, some participants wanted to know some information about the characters’ values, for who they may be fighting for, and the origin of the hapu (clan) of characters. It was also thought that being more formally introduced to the characters could help make them seem “less like strangers”.

Because you could say which tribe you are from and then pick your tribe and stuff and different tribes have a different dude or something (comment during discussion about what is seen as culturally applicable).

Group 7 Male Participant 1

The Guide could be a role model for taitamariki. Participants acknowledged the Guide could be utilized as a role model for taitamariki.

Actually one of my mates has stopped himself from being depressed and one of the reasons why he killed himself was his family was too hard to get to and there was nothing to help him on his journey. I think some young people find it hard to talk to their parents...If we had a role model, we would go straight to them. But some young people don’t really have role models in terms of what they want to do. But not many people have people to look up to, to help them on the way and maybe the video game (SPARX) would be something to help them along. And maybe at the end of the game you could guide them to actually go and talk to their parents or wherever they are living and talk to them. Get them to sit down and help them do what they want (a spontaneous response nearing the end of the focus group from a young person that had been listening, but not saying much throughout the group).

Group 3 Male Participant 1

### Whānau Are Important for Young People’s Well-Being

Whānau expressed a range of opinions about when they needed to be informed that their taitamariki were using SPARX. Whānau thought that they would benefit from having resources to support them while their taitamariki were using SPARX. There were also differing opinions about where whānau would want their taitamariki to be using SPARX.

Some feelings from parents about the inclusion of whānau in the process varied. Some parents expressed that they wanted to know more about SPARX before their taitamariki used the program, while other whānau preferred to be informed when their taitamariki were either using SPARX or had completed it. Some parents were comfortable with not being informed, as long as their taitamariki could talk to someone about their depression and get the support they needed, such as from extended whānau or a clinician.

I am very much a traditionalist, and if it was my child, I would like to know before they got onto the computer that they were going through this type of depression...I would prefer some kind of means of being able to identify that there is a problem and actually being with them, right beside them, as they work through it. So if they get an opportunity to sit on the computer on their own then I am aware of that, but I wouldn’t want them to spill their feelings to a computer first. I would be very hurt if that was my child (comment during spontaneous discussion about how whānau can support their youth).

Group 3 Male Parent 1

The resources that could be used for whānau to help them understand about depression were discussed. There was a range of opinions about how SPARX could be used with whānau members. However, most participants in the focus groups agreed that it was important to think about extra resources that whānau could use to support them while their taitamariki completed the SPARX program. This could include having an extra module within SPARX that was applicable to whānau members. Alternatively, a booklet could be developed that provided psychological education to whānau about depression.

I think you should have another disc (SPARX) for the parents... something anyway just so then they can help the child through that (in response to MS asking about what resources are needed for the treatment of depression).

Group 6 Female Participant 1

The range of location sites for use of SPARX by taitamariki is noted. Whānau were open to a range of different localities where their taitamariki could be using SPARX. This varied from SPARX being used at a high school, a library, a health service, or in the whānau home.

If they want to get dropped off at the library that is fine, if they want to do it in their room and close the door, that’s fine. If they want me out of the house, that’s fine (in response to MS asking about what resources are needed for the treatment of depression).

Group 3 Male Parent 1

### Ideas for Improvement of Smart, Positive, Active, Realistic, X-Factor Thoughts for Māori

Participants suggested that SPARX needed more activities for Māori males, and that the puzzles and challenges needed to be more difficult. SPARX also needed to include language that reflected taitamariki understandings about depression.

SPARX needs activities that appeal to male adolescents. These added activities would need to direct male adolescents to participate in physical challenges such as fishing, skate boarding, kapa haka, or mau rākau (traditional Māori martial arts). The reasons given were that SPARX contains a lot of text, and male adolescents may not want to sit down at a computer and read a lot of text.

I think it (SPARX) is pretty good, but I would probably get lost on the computer because I don’t know if many Māori boys or the other kids like using computers because I definitely don’t and I get lost straight away and I just think I would just sit there. I don’t want to just sit there and watch and listen to video games or something. Maybe SPARX needs relaxing things like fishing or something or more activities to do in the game (in response to MS asking what would be good about using a program like SPARX for young people with low mood).

Group 3 Male Parent 1

The use of language in SPARX for adolescents may not be adequate. Participants thought that adolescents do not always have the necessary language to be able to identify that they may be experiencing depression. Adolescents may need education about what terms to use to describe how they are feeling. Conversely, it was thought that mental health professionals needed to listen more carefully to the colloquialisms of adolescents and then use these in their clinical practice.

You know what you were talking about with regard to the language, how you need to teach young people a whole new language, maybe it is the other way round. Maybe it is them teaching us the language and then us interpreting it (spontaneous discussion about what else SPARX may need to be maximally useful for young people).

Group 7 Female Parent 1

SPARX has too much text to read for some participants. A majority of participants indicated that the text needed to be shortened so that it was more manageable to read.

Does it read alright the text?

First Author, MS

Yes, it does. It is just a lot of words. And it could probably be shortened or you could get straight to the point and maybe the facts are at a different stage or before something.

Group 6 Female Participant 1

Developers should include the Māori language within SPARX. Even though SPARX was designed as a program for all ethnicities, some of the participants stated that it was important to incorporate Māori language in the text as a way to help taitamariki to connect and have ownership of the SPARX program.

It might just give it a little bit of ownership back to those young people (to have Māori terms in the text), this is my language...It is not an American thing; it is actually a Māori thing or a New Zealand thing (in response to MS asking would it be helpful if there were Māori words in SPARX).

Group 7 Female parent 1

Developers should increase the use of audio rather than text. The audio clips were thought to help support those taitamariki who struggled with the amount of text or who simply do not like to read.

What if you had a voice over instead of the writing?

Group 2 Female Participant 3

Yes, that is another good point. We are going to do that.

The First Author, MS

Not many people our age like to read.

Group 2 Female Participant 3

I don’t like reading (spontaneous discussion about how there was too much text in the prototype of SPARX).

Group 2 Female Participant 1

### Focus Group Questionnaire

Nineteen of the 26 participants completed a questionnaire. These results demonstrated that; most participants were able to express their opinions in the group; most people liked the graphic style of SPARX; the majority liked the content of SPARX; and most liked the cultural content of SPARX. In Table 3, the Likert rating scale ranged from 1 to 5, 5 = highest rating.

**Table 3:** Results of focus group questionnaire.

Number of participants	Questions	Mean	SD
n=19	How much were you able to express your opinions in the focus group?	3.95	1.17
n=19	Overall what did you think about the look and style of the game?	4.2	0.8
n=19	Overall what did you think about the content (messages and information to help people) in the game?	4.00	0.88
n=19	Overall what did you think about the cultural content in the game (Māori costume design and building/environment) designs?	4.2	0.6

## Discussion

### The Participants’ View

This is a unique study, as it is the first, to our knowledge, to gather the opinions of ethnic minority indigenous people about their experiences of a computerized therapy program and to do so using a research methodology developed by Māori people to ensure good outcomes for Māori people participating in research. Participants were positive about the prototype version of SPARX, and their views were used to refine and improve on the final version of SPARX. Cultural relevance was viewed as being important for the engagement with SPARX among Māori young people. The incorporation of Māori symbols and the use of a Māori actor to provide the voice over for the character of the Guide all led to acceptance of SPARX by Māori, and their inclusion was seen as key in the dissemination of the SPARX program. Participants’ highlighted specific opportunities to improve SPARX for Māori, and the ideas from these focus groups were incorporated into the final design of SPARX. Participants also highlighted specific opportunities to improve SPARX for Māori.

Participants’ thought that learning a simple relaxation exercise was particularly beneficial, thus indicating that some skills can be learned without the aid of a therapist. This finding is in keeping with the literature [37], which purports that cCBT programs without direct therapist input can teach skills. This has implications for primary care settings and school environments where CBT expertise is not always available, but where programs like SPARX could make evidence-based interventions accessible to a wider client group.

Although SPARX was designed to appeal to more than one cultural group, SPARX does contain: Māori specific artwork; Māori-based characters; some Māori language; and the Guide character (who is the virtual therapist and main character, after the user’s avatar) has a distinct Māori-English accent. Feedback from the focus groups confirmed the importance of the cultural relevance of the design. Most of the participants noticed the Māori designs within the SPARX environment and thought the designs enhanced the engagement with the program. Culturally adapted mental health interventions, targeted to a specific cultural group, are thought to be approximately four times more effective than interventions provided to groups from a variety of cultural backgrounds [38].

Māori, like other ethnic groups, are not homogeneous. The SPARX prototype was not, and perhaps cannot be, applicable to all Māori. Durie [26] emphasizes this point when asserting that one approach does not fit for all Māori.

The graphic designs in SPARX represented a “leap forward” in terms of contemporary tikanga (Māori protocols) employed within modern game design, and this process has provided some much needed information about the process of adapting nonindigenous interventions for use by indigenous people, about the efficacy of these interventions for indigenous people, and, more pointedly, for Māori [19].

Whānau are important for the well-being of young people [39]. This study is the first to take these opinions into account in the development of a cCBT program, to our knowledge. Whānau noted the cultural designs and thought it helped to engage taitamariki with the SPARX resource. Family members were keen to be involved in the lives of their young people and to have the resources to help them support their young people.

Indigenous minority adolescents often do not access help for depression [13]. The flexibility and privacy of cCBT, and the ability to design computerized interventions that look appealing to indigenous young people, may help reduce some of the barriers these young people face in accessing help. In addition, this approach could support those whānau and taitamariki who live in remote areas [40].

### Strengths

In this study, we gathered views from young people and their families from an indigenous ethnic minority group, a group frequently neglected in trials of therapeutic interventions. In New Zealand, there has been an attempt to develop a social policy about whānau ora (healthy families) [41]. The SPARX resource, and the approaches taken in its development, may be one particular way to help achieve whānau ora.

Based on the results of this study, we were able to improve SPARX for Māori; for example, we included audio files for the text wherever possible as a direct result of the feedback obtained from participants in this study.

### Limitations

This is a small study, limited to focus groups and one individual interview. The views, therefore, are not reflective of all Māori. Conducting individual interviews may have provided a greater range of in-depth opinions; however, focus groups allowed for richness in interaction between participants, which individual interviews would not have provided. The findings of this study were not based on a clinical population. We thought that within this minority indigenous population depression is common and help seeking is low.

Therefore, we did not want to create a barrier to Māori peoples’ participation in this study.

### Implications for Computerized Cognitive Behavioral Therapy Research and Delivery

It is important for researchers to consult with indigenous groups when developing programs for these young people. These processes can then lead to greater engagement with the specific program. Once a program has been developed, it is essential that support be provided to indigenous families to help families encourage and support their young people with depression when using cCBT. Traditional Māori (and other indigenous) families will want to be a part of how their young person engages with a cCBT self-help resource, and they will want to know when and where their young people are using it. Hence, family resources need to be developed alongside a cCBT program so that information is provided to the family about what program their young person is using. This collectivist approach to cCBT and its delivery contrasts considerably with the often individualistic focus of cCBT delivery to date.

### Conclusions

In general, taitamariki and whānau supported the contemporary tikanga approach to the graphic designs that were used for the characters and environment within SPARX. These findings are important, as a resource like SPARX, which has sought to engage indigenous youth in its creation (and subsequent formal evaluation), has never been developed previously. This study provided information that was utilized in the further refinement and development of SPARX to help ensure maximal applicability to taitamariki and provides a potential model for other cCBT interventions.

## Abbreviations

CBT: cognitive behavioral therapy
cCBT: computerized CBT
GIA: General Inductive Analysis
RADS-SF: Reynolds Adolescent Depression Scale-Short Form
SPARX: Smart, Positive, Active, Realistic, X-factor thought
